# Supplementary material for: The unprecedented 2015/16 Tasman Sea marine heatwave
Source: Nat Commun. 2017 Jul 14;8:16101. doi: 10.1038/ncomms16101 (PMC5519980; doi:10.1038/ncomms16101)
Supplement: Supplementary Information [file ncomms16101-s1.pdf]

File Name: Supplementary Information
Description: Supplementary Figures and Supplementary Tables

File Name: Peer Review File
Description:

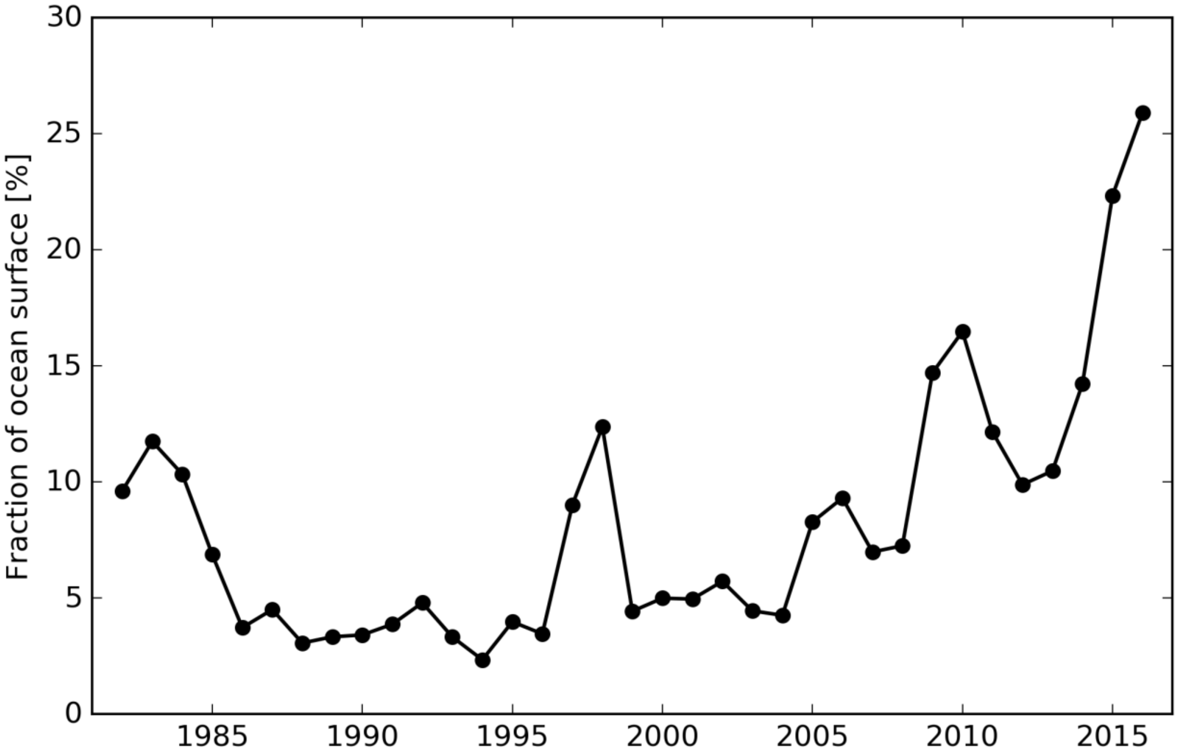

Supplementary Figure 1. Proportion of ocean surface covered by a marine heatwave event that was the largest on record according to any of the four MHW metrics (duration, maximum intensity, mean intensity, cumulative intensity) for events occurring during each year from 1982 to 2016.

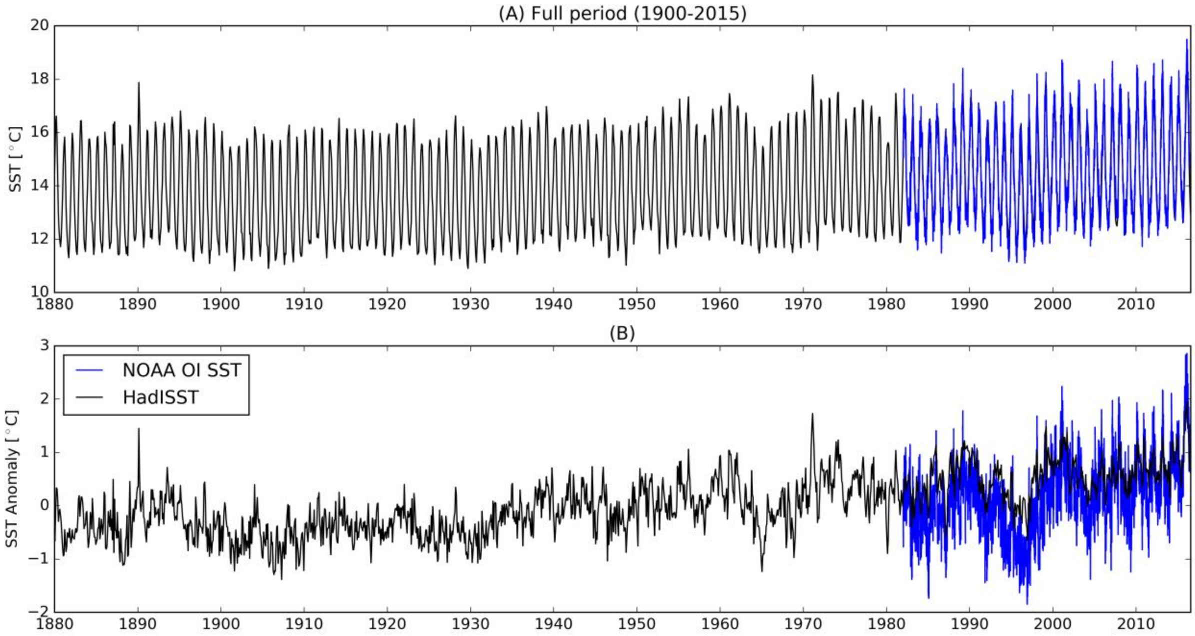

Supplementary Figure 2. Time series and properties of sea surface temperatures off southeast Australia (SEAus). Shown are time series of (a) sea surface temperatures (SSTs) and (b) SST anomalies averaged over the SEAus region since 1880 from HadISST (black lines) and since 1982 from NOAA OI SST (blue lines).

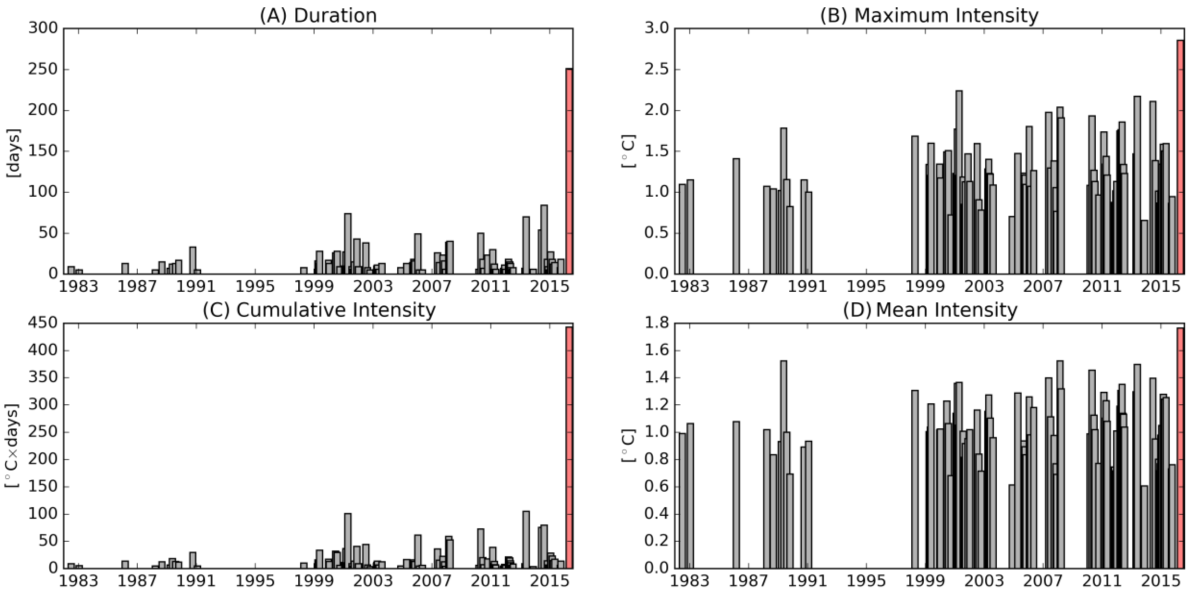

Supplementary Figure 3. Properties of all detected marine heatwave events in the southeast Australia region (SEAus). The (a) duration, (b) maximum intensity, (c) cumulative intensity, and (d) mean intensity are shown as bars for all events in the SEAus region back to 1982. The 2015/16 event is shown by a red bar.

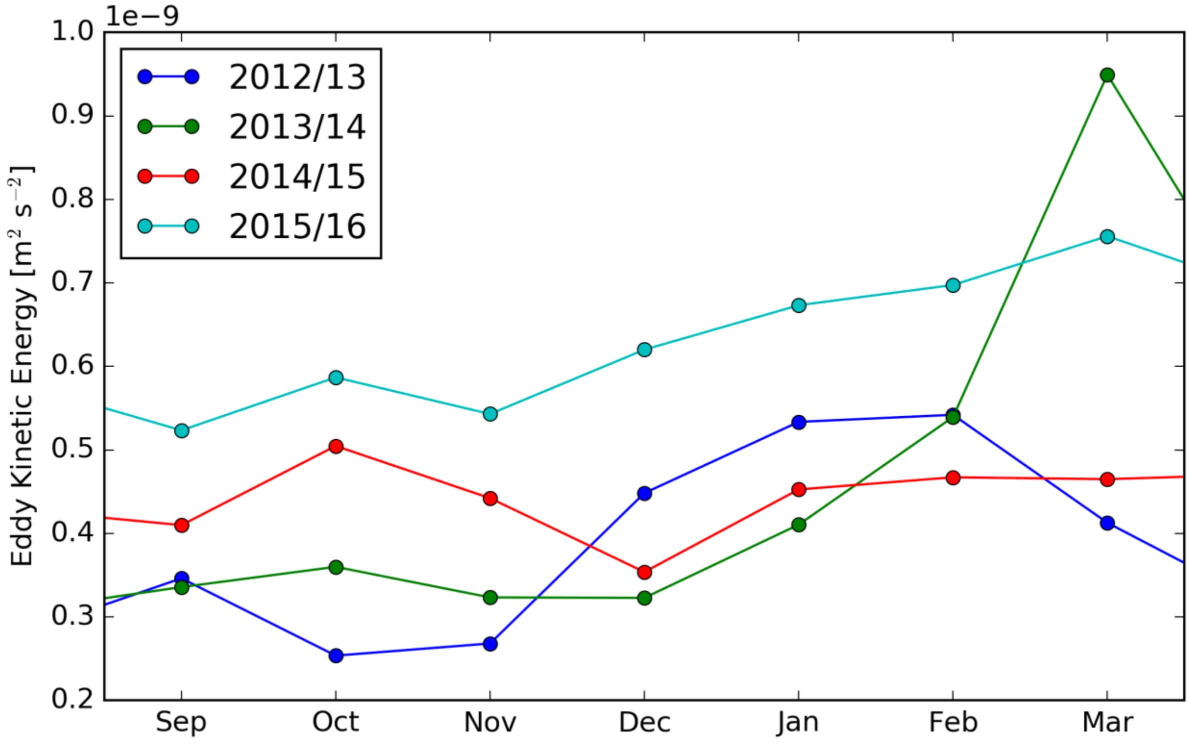

Supplementary Figure 4. Eddy kinetic energy (EKE) over the southeast Australia (SEAus) region. EKE was calculated from surface geostrophic currents (OceanCurrent data product), after removing the seasonal climatology, and then averaged over the SEAus region and within each month over the 2012-2016 period. Shown here are the September through March (following year) periods for 2012/13 (blue), 2013/14 (green), 2014/15 (red), and 2015/16 (cyan).

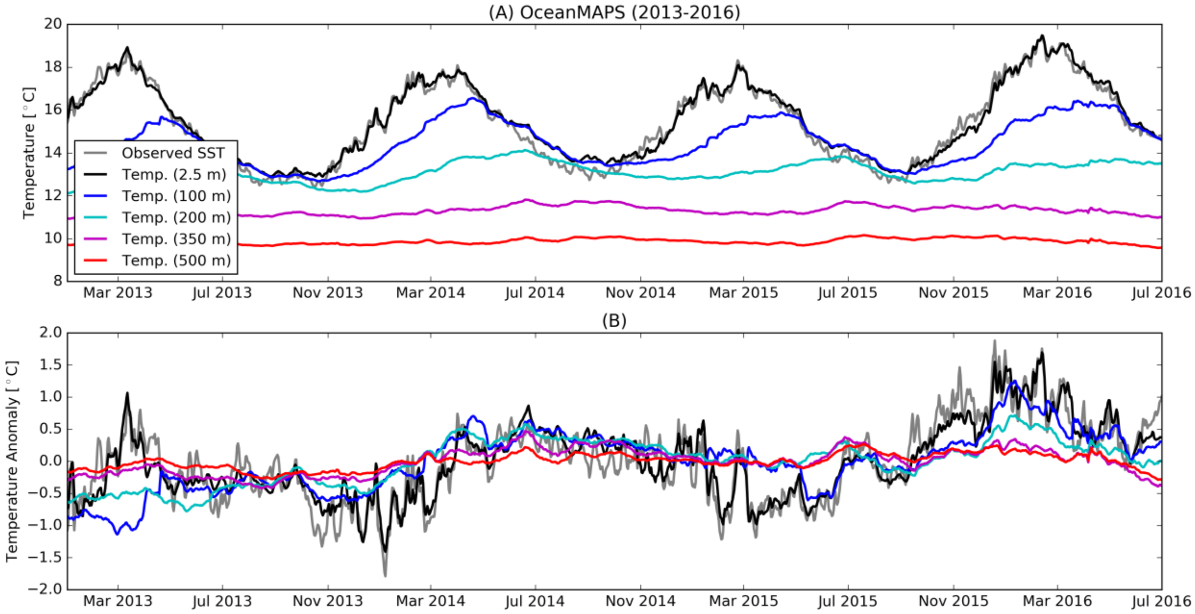

Supplementary Figure 5. Time series of OceanMAPS temperatures off southeast Australia (SEAus). Shown are time series of (a) absolute temperatures and (b) temperature anomalies averaged over the SEAus region since 2013 from NOAA OI SST (grey lines) and OceanMAPS at 2.5 m depth (black lines), 100 m depth (blue lines), 200 m depth (cyan lines), 350 m depth (magenta lines) and 500 m depth (red lines).

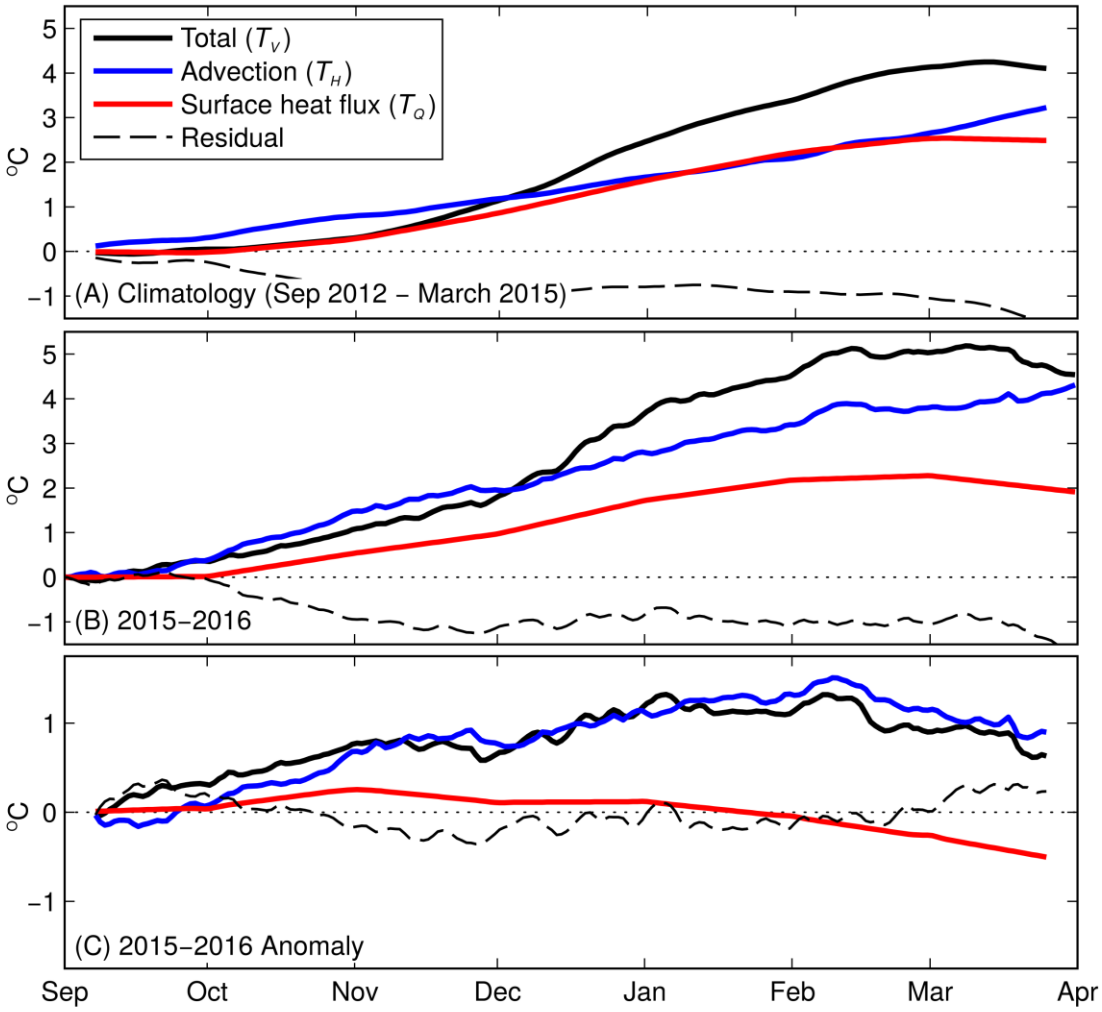

Supplementary Figure 6. Temperature budget during the 2015/16 marine heatwave, as in Fig. 6 but using GODAS air-sea heat flux rather than NCEP CFSv2.

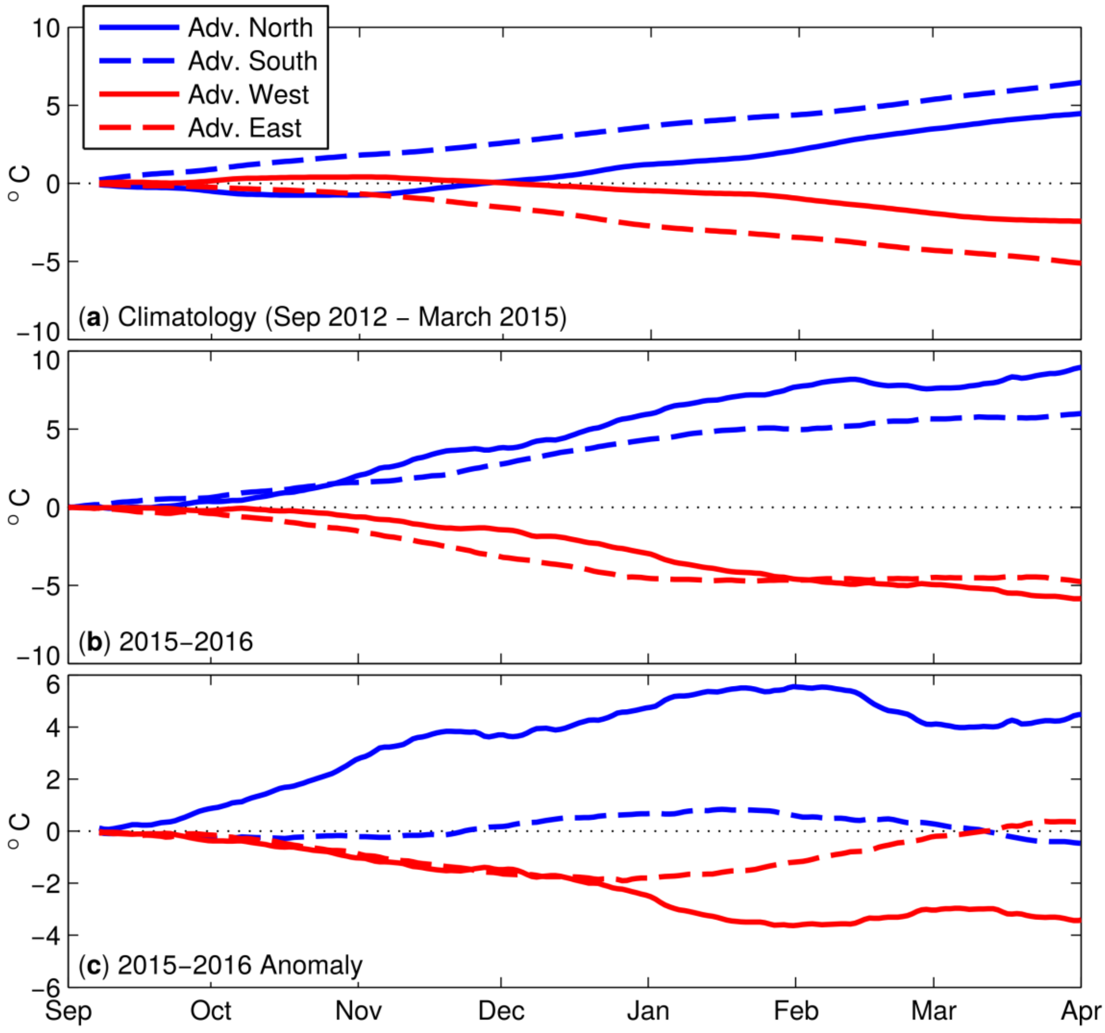

Supplementary Figure 7. Decomposition of horizontal advection terms in the temperature budget (see Fig. 6) during the 2015/16 marine heatwave. (a) The climatology of the contributions due to horizontal advection across the north (solid blue line), south (dashed blue line), west (solid red line) and east faces (dashed red line). (b) The contributions of these four terms, as in (a), but for 2015-2016 only. (c) The anomalous contributions during 2015-2016, calculated as the difference between panels (b) and (a). Note that positive values indicate temperature advection into the box (increasing the temperature) and negative indicate temperature advection out of the box (decreasing the temperature). The sum of the four components is equal to the horizontal advection contribution (Fig. 6, blue lines).

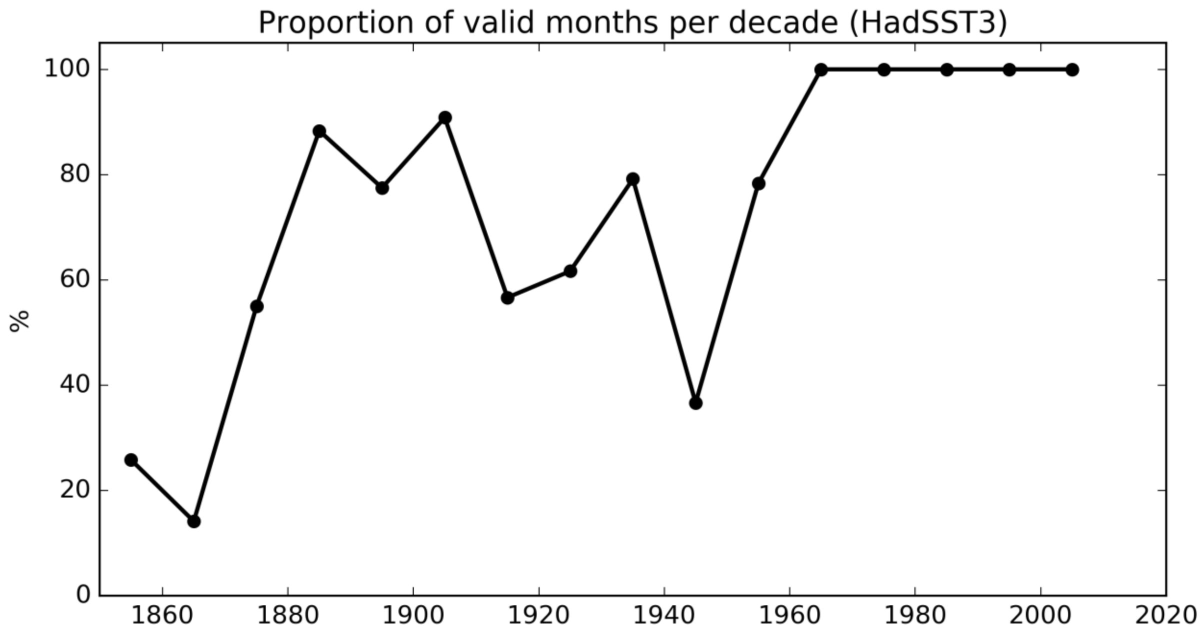

Supplementary Figure 8. Proportion of valid monthly data from HadSST3, averaged over the SEAus region, shown as decadal blocks from 1850 to 2010.

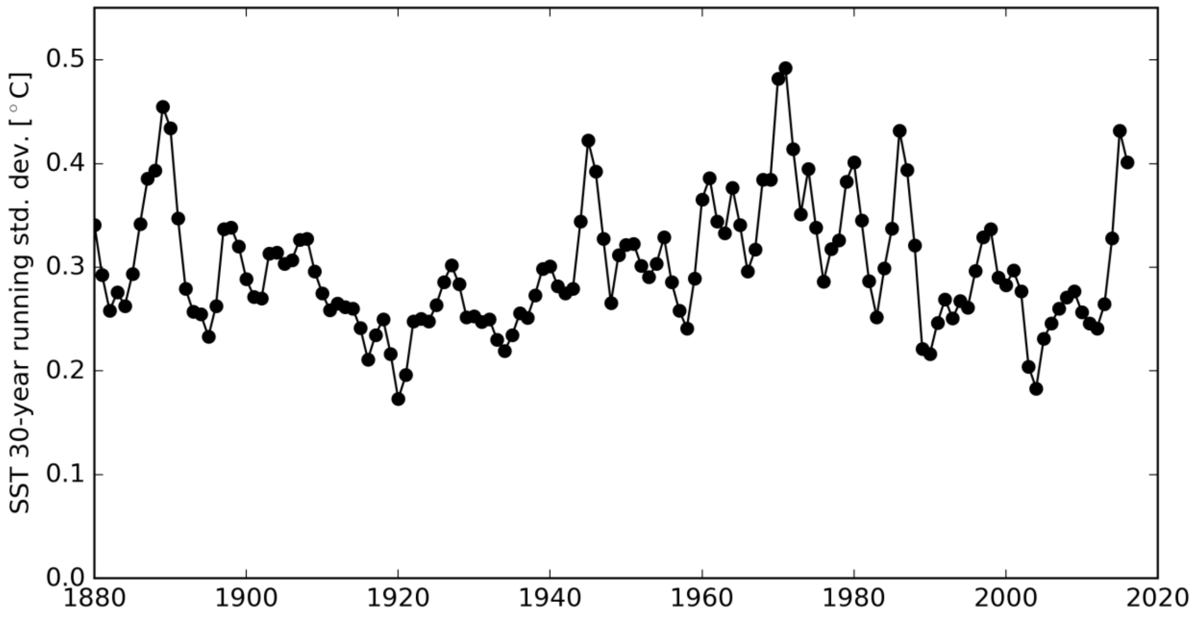

Supplementary Figure 9. Standard deviation of HadISST, averaged over the SEAus region from 1880-2016, in running 30-year blocks.

Supplementary Table 1. Near-shore sub-surface temperature logger sites along the east coast of Tasmania. “Complete (%)” indicates the proportion of valid daily data in each time series as a percentage.

Site	Start date	End date	Latitude (°S)	Longitude (°E)	Depth (m)	Complete (%)
George III Reef	28/5/2004	20/5/2016	43.51	146.98	10.2	88
Cape Peron	4/5/2005	18/4/2016	42.74	148.01	12.0	99
Schouten Island	16/1/2007	15/4/2016	42.28	148.29	12.0	79
Mouldies Hole	26/2/2005	14/4/2016	43.59	146.92	6.0	74
One Tree Point	28/9/2004	20/5/2016	43.11	147.39	8.0	99
Maria Island	31/7/2008	6/3/2017	42.59	148.23	19.0	91
Wineglass Bay	22/1/2014	15/4/2016	42.17	148.31	12.0	97
Coles Bay	16/1/2007	15/4/2016	42.13	148.30	6.0	98
Magistrates Point	23/6/2004	22/6/2016	42.58	148.05	10.1	73
Iron Pot	6/12/2006	20/5/2016	43.05	147.42	9.0	99
Wedge Island	18/5/2005	20/5/2016	43.13	14768	10.2	92
Swansea	5/5/2005	15/4/2016	42.12	148.09	8.4	58
Bicheno	4/6/2005	5/5/2016	41.87	148.31	13.0	99

Supplementary Table 2. CMIP5 models used in this event attribution analysis. The number of ensemble members are indicated for each model and experiment considered. The bias correction is the ratio of the historical runs non-seasonal standard deviation to the observed non-seasonal standard deviation, averaged across all runs for each model, and was used to bias-correct all runs from that model.

**Model**	**Historical**	**HistoricalNat**	**RCP8.5**	**Bias correction**
ACCESS1.3	3	3	1	1.32
CanESM2	1	3	5	1.10
CSIRO Mk3.6.0	10	10	10	1.42
CNRM-CM5	1	5	5	0.80
HadGEM2-ES	4	4	4	0.96
IPSL-CM5A-LR	6	3	4	0.98
IPSL-CM5A-MR	3	3	1	0.91
**Total**	**28**	**31**	**30**	-
